# GLP-1 Improves Adipocyte Insulin Sensitivity Following Induction of Endoplasmic Reticulum Stress

**DOI:** 10.3389/fphar.2018.01168

**Published:** 2018-10-16

**Authors:** Yaojing Jiang, Zhihong Wang, Bo Ma, Linling Fan, Na Yi, Bin Lu, Qinghua Wang, Rui Liu

**Affiliations:** ^1^Department of Endocrinology, Huashan Hospital, Fudan University, Shanghai, China; ^2^Department of Obstetrics and Gynecology, Tianjin Central Hospital of Gynecology Obstetrics, Tianjin, China; ^3^Division of Endocrinology and Metabolism, Keenan Research Centre for Biomedical Science of St. Michael’s Hospital, University of Toronto, Toronto, ON, Canada

**Keywords:** glucagon-like peptide-1, insulin sensitivity, adipocyte, endoplasmic reticulum stress, mTOR

## Abstract

Glucagon-like peptide 1 (GLP-1) improves insulin resistance of adipose tissue in obese humans. However, the mechanism of this effect is unclear. Perturbation of endoplasmic reticulum (ER) homeostasis impairs insulin signaling. We hypothesized that GLP-1 could directly improve insulin signaling in ER-stressed adipocytes. Here, we examined the effects of GLP-1 on ER stress response in fat cells in an obese and insulin-resistant murine model. We found that GLP-1 analog liraglutide reduced ER stress related gene expression in visceral fat cells accompanied by improved systemic insulin tolerance. Consistently, GLP-1 decreased CHOP expression and increased insulin stimulated AKT phosphorylation (p-AKT) in thapsigargin, a ER stress inducer, treated white fat cells differentiated from visceral stromal vascular fraction. We further found blocking CHOP expression increased insulin stimulated p-AKT in ER-stressed fat cells. Of note, we found mTOR signaling pathway contributed to the expression of ATF4 and subsequently the CHOP expression in ER stress response, while GLP-1 inhibited mTOR activity as exemplified by elevated autophagosome formation and increased LC3II/LC3I ratio. These findings suggest that GLP-1 directly modulates the ER stress response partially via inhibiting mTOR signaling pathway, leading to increased insulin sensitivity in adipocytes.

## Introduction

Intestinal hormone glucagon-like peptide 1 (GLP-1) potentiates insulin secretion in a glucose-dependent manner via activating GLP-1 receptor (GLP-1R) highly expressed on islet β cells (Drucker, 2006). GLP-1R was also widely expressed on non-β cells and exerts metabolic functions including suppression of glucagon secretion, repression of appetite and slowdown of gastric emptying. Of note, GLP-1 has shown functions in improvement of insulin resistance (IR) in insulin responsive tissues especially adipose tissue in obese and diabetic animals and humans (Gastaldelli et al., 2016). However, the mechanism of the effects of GLP-1 on insulin signaling in adipocytes is still unclear.

Previous work from our group and others has amplified GLP-1R transcript in differentiated 3T3-L1 cell and stromal vascular fraction (SVF) of murine adipose tissue (Liu et al., 2013, 2016). GLP-1R was also found in adipocytes and the SVF of human visceral adipose tissues using RT-PCR and immunohistochemistry (Vendrell et al., 2011). However, since insulinotropic and weight loss action associated with GLP-1R agonism improves insulin sensitivity in animals and humans, evidence supporting that GLP-1 exerts direct effects on adipocyte functions is limited. Recently, Gastaldelli et al. (2016) evaluated the acute effects of GLP-1R agonists on adipose IR in overweight and obese subjects with newly diagnosed impaired glucose tolerance during a glucose load. They found GLP-1R agonists reduced adipose IR and enhanced the antilipolytic effect of insulin (Gastaldelli et al., 2016), suggesting that GLP-1 improved insulin signaling in stressed adipocytes via directly activating GLP-1R expressed on adipocyte.

Chronic overfeeding induces adipocyte hypertrophy which is a stress condition for the endoplasmic reticulum (ER). To ameliorate ER stress and ensure correct protein folding, the ER acutely activates the unfolded protein response (UPR). UPR signaling is mediated through three parallel pathways, including PERK, Ire1, and ATF6 (Wu and Kaufman, 2006). Activation of PERK phosphorylates the translation initiation factor eIF2α, which transiently attenuates global protein synthesis, reducing the load on the ER. Meanwhile, eIF2α phosphorylation leads to enhanced translation of ATF-4, which then upregulates the expression of a transcriptional repressor CAAT/enhancer binding protein homologous protein-10 (CHOP10). CHOP reduced the Akt activation and subsequently affect insulin signaling (Maris et al., 2012). Activation of Ire1 and ATF6 splices XBP-1 mRNA and ATF6 protein respectively leading to generation of active transcription factors, which are involved in the activation of genes encoding molecular chaperones (Salvado et al., 2015).

Although GLP-1R agonist increased insulin-stimulated glucose uptake in fat derived cells under normal condition (Idris et al., 2002), whereas the clinical indications of GLP-1 were obesity and obesity-related type 2 diabetes. Therefore, in the present study, we investigated whether GLP-1 was able to improve adipocyte insulin sensitivity under ER stressed condition. Our results show that GLP-1 improved the insulin signaling via suppressing the expression of CHOP downstream of the PERK arm of UPR, providing a potential mechanism for adaptation to metabolic and cellular stress.

## Materials and Methods

### Materials

Cell culture reagents, DNase I and TRIzol, were purchased from Life Technologies (Carlsbad, CA, United States). GLP-1, thapsigargin, tunicamycin, isobutylmethylxanthine (IBMX), dexamethasone (Dex), and 4′,6-diamidino-2-phenylindole (DAPI) were acquired from Sigma Chemical (St. Louis, MO, United States). Complete Protease Inhibitor Cocktail was purchased from Roche Applied Science (Mannheim, Germany). TaKaRa PrimeScript^TM^ RT reagents kits and TaKaRa SYBR premix Ex Taq were acquired from TaKaRa Bio (Kyoto, Japan). Antibodies for LC3, ATF-4, CHOP, phospho-AKT Ser47 (p-AKT), phospho-mTOR Ser2448 (p-mTOR), phospho-eIF2a Ser51 (p-eIF2a), phospho–PERK Thr980 (p-PERK), b-tublin, and FITC-conjugated anti-rabbit and anti-mouse IgG antibodies were purchased from Cell Signaling Transduction (Boston, MA, United States). Antibodies for GAPDH were acquired from Santa Cruz Biotechnology (Santa Cruz, CA, United States). Lipofectamine2000 and siRNA control were purchased from Life Technologies (Grand Island, NY, United States). All other chemicals of analytical grade were acquired from Dingguo Bio (Shanghai, China).

### Animal Studies

Six-week-old male ob/ob (BKS.Cg-m+/+Leprdb/J) (Slac Laboratory Animal, Shanghai, China) mice were housed under controlled temperature conditions and a 12-h light/12-h dark with free access to food and water except where noted. All animal experiments were carried out in accordance with protocols and guidelines approved by the Institutional Animal Care and Use Committee. Commencing at 6 weeks of age, when all mice were normoglycemic, they were given once-daily intraperitoneal (i.p.) injections of Liraglutide (100 μg/mouse) or phosphate-buffered saline (PBS) for 2 weeks and then sacrificed. Considering the repressive effect of liraglutide on food intake, we feed all mice with fixed amount of food (2 g per mice per day) from day 7 to day 14, according to the amount of food consumed by liraglutide-treated mice during the first week. Body weight and random blood glucose were measured.

### Intra Peritoneal Glucose Tolerance Test (IPGTT)

For IPGTT, mice were fasted for 14 h and then given 2 g glucose/kg body weight via intra-peritoneal injection. Blood was drawn from the tail vein at 0, 15, 30, 60, and 90 min after glucose administration and glucose level were measured by a glucometer (Elite, Bayer).

### Intra Peritoneal Insulin Tolerance Test (IPITT)

For IPITT, mice were fasted for 5 h and then given 1.0 unit insulin/kg body weight via intra-peritoneal injection. Blood was drawn from the tail vein at 0, 15, 30, 60, and 90 min after insulin administration and glucose level were measured by a glucometer (Elite, Bayer).

### Isolation of Stromal Vascular Fractions and *in vitro* Differentiation

Induction of adipocytic differentiation from SVFs was performed as described previously (Xue et al., 2015). Briefly, visceral adipose tissue fragments were minced from 6-week-old male Sprague-Dawley rats and digested with 1 mg/ml collagenase type I. After centrifugation, the pellets were re-suspended and filtered through a 100 mm strainer. The stromal-vascular cells suspension was plated in tissue culture flasks with high-glucose DMEM containing 10% fetal bovine serum and antibiotics. 24 h later, cells were washed with PBS and adherent cells were maintained until the monolayer of cells reached confluence. Confluent cells were induced with a medium containing 10% FBS, 5 μM insulin, 0.5 mM isobutylmethylxanthine, 2 μM dexamethasone and 125 μM indomethacin. After 48 h, cells were cultured with a medium with 1 μM insulin for 3 days. After this period, cells were grown for another 3–6 more days in culture medium until at least 95% of the cells had accumulated fat droplets.

### 3T3-L1 Cell Culture and Differentiation

3T3-L1 pre-adipocytes were cultured as previously described (Liu et al., 2013). Briefly, cells were maintained in high glucose DMEM containing 25 mM glucose, 10% fetal bovine serum and antibiotics, and confluent pre-adipocytes were grown for another 2 days in culture medium supplemented with 1 μM insulin, 0.5 mM IBMX, and 0.1 μM Dex (MDI) and for further 2 days in culture medium with 1 μM insulin. After this period, 3T3-L1 cells were grown for 3–6 more days in culture medium, after which at least 95% of the cells had accumulated fat droplets.

### RNA Extraction and Quantitative Real-Time PCR

Total RNA was extracted using TRIzol reagent. After the RNA was treated with DNase I, 1 μg of RNA was reverse-transcribed using TaKaRa PrimeScript^TM^ RT reagents kits, according to manufacturer’s instructions. Quantitative real-time polymerase chain reaction (qPCR) was performed with ABI Prime 7500. Samples were prepared using TaKaRa SYBR premix Ex Taq, according to the manufacturers’ instructions. Each PCR product was verified for its single amplification by melting curve analysis. The gene-specific primers for amplification are listed in **Table 1**. The transcript expression levels were normalized to the expression of GAPDH.

**Table 1:** Forward and reverse primers used for qPCR.

	Forward	Reverse
BIP	GTTTGCTGAGGAAGACAAAAAGCTC	CACTTCCATAGAGTTTGCTGATAAT
CHOP	GTCCAGCTGGGAGCTGGAAG	CTGACTGGAATCTGGAGAG
ATF4	GGACAGATTGGATGTTGGAGAAAATG	GGAGATGGCCAATTGGGTTCAC
sXBP1	CTGAGGTCCGCAGCAGGT	TGTCAGAGTCCATGGGAAGA
tXBP1	AGCAGCAAGTGGTGGATTTGGAAG	AAGAGGCAACAGTGTCAGAGTCCA
Atg7	ATGGGGGGACCCTGGACTG	CAGAACAGTGTCGTCATC
p62	TGGGCAAGGAGGAGGCGACC	CCTCATCGCGGTAGTGCGCC
b-Actin	TGTGACGTTGACATCCGTAAAGAC	TCCACACAGAGTACTTGCGCTC

### RNAi Experiment

Three target regions (32:858-882, 33:1378-1402, and 34:1507-1531) for ATF4 were selected and synthesized by QIANGEN (Germany). A scrambled fragment 5′-AAGAGGAGCATATTGGGAAGA-3′ was used as a negative control. Transfection of siRNA into 3T3-L1 cells was performed with Lifectamine 2000 according to the manufacturer’s instruction.

### Western Blot Analysis

Following drug treatment as indicated, cells were lysed in RIPA buffer (1% Nonidet P-40, 0.5% sodium deoxycholate, and 0.1% SDS in PBS) supplemented with protease and phosphatase inhibitor mixtures. Forty to fifty micrograms of cell lysate protein was resolved by SDS-PAGE and immunoblotted as described previously (Liu et al., 2014). The proteins were transferred to PVDF membrane and immunoblotted with primary antibodies overnight. Specifically bound primary antibodies were detected with horseradish peroxidase (HRP)-coupled secondary antibody and enhanced chemiluminescence.

### Immunohistochemisry

For histochemistry analysis, white adipose tissues were isolated, and the tissue fixation and paraffin-embedding were conducted as described previously (Soltani et al., 2011). Sections were stained with anti-CHOP/GADD153 antibody. FITC-conjugated anti-mouse IgG antibody was added, followed by nuclear DAPI counterstaining. Images were obtained using a Nikon fluorescence microscope.

### Statistical Analysis

The values are presented as means ± SEM. Comparisons between groups were performed with Student’s unpaired *t*-test and, in cases of multiple time points and treatments, by one-way ANOVA. *P*-values < 0.01 were considered to be highly significant and < 0.05 were considered to be significant.

## Results

### GLP-1 Reduces ER Stress and Improves Insulin Resistance in Adipose Tissue of ob/ob Mice

GLP-1R agonists have previously been shown to either prevent or ameliorate experimental obesity and preserve insulin sensitivity in multiple preclinical models. Accordingly, we examined glucose control, adipose histology, and the expression of markers important for the response to ER stress in obese ob/ob mice treated with vehicle alone or with GLP-1 analog liraglutide. Body weight at the end of the 2 weeks treatment period were 47.85 ± 0.9 versus 45.45 ± 0.67 for PBS versus liraglutide treated mice, *P* = 0.0534. Blood glucose levels at the end of the treatment were 9.07 ± 0.65 versus 7.52 ± 0.45 for PBS versus liraglutide treated mice, *P* = 0.0192.

**FIGURE 1:**
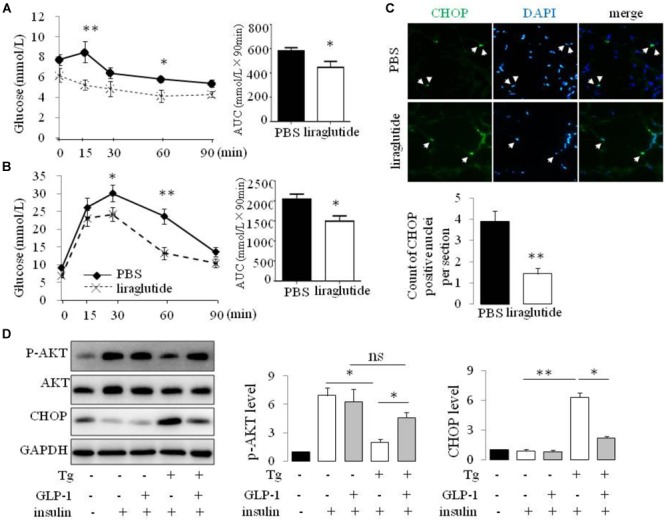
GLP-1 reduced the expression of makers of ER stress in primary rat adipocytes subjected to ER stress and improves insulin sensitivity of ob/ob mice. ob/ob mice (6 weeks old) were given once-daily i.p. injection of PBS or liraglutide for 2 weeks. Blood glucose level in PBS- and liraglutide-treated mice were determined during IPITT **(A)** and IPGTT **(B)**. Glucose responsiveness of the corresponding experimental groups was shown as a measurement of glucose area under the curve (AUC) of the IPGTT or IPITT graph. Data represent means ± SE of 6 mice per group. **(C)** Photomicrographs of representative adipose CHOP staining (arrows point to CHOP-positive nuclei) in PBS- and liraglutide-treated mice. Magnification, 200×. The numbers of CHOP-positive adipocyte were calculated in a total of 21 microscopical sections from three mice per group. Data represent means ± SE. **(D)** Fat pad from two rats was pooled for SVF isolation. Adipocytes differentiatied from SVF were fasted for 6 h in low-glucose DMEM media with 0.2% BSA (fasting media) and then were exposed to either vehicle alone or 5 μM thapsigargin (Tg) in fasting media for 16 h in the absence or presence of 50 nM GLP-1. The cells were then stimulated with insulin (100 nM) in fasting media for 10 min. Two wells of cell extracts were pooled together and were analyzed by immunoblotting for phospho-AKT Ser47 (p-AKT), total AKT, CHOP, and GAPDH (loading control). Results are means ± SE of four independent experiments. ^∗^*P* < 0.05 and ^∗∗^*P* < 0.01.

Liraglutide treatment improved insulin tolerance (**Figure 1A**) and glucose tolerance (**Figure 1B**) in ob/ob mice. The transcription factor CHOP serves a number of important cellular functions and has also been implicated as a critical component of the ER stress response (Oyadomari and Mori, 2004). We detected an increase in CHOP-positive nuclei in visceral adipocytes comparing with epididymal adipocytes from ob/ob mice. Furthermore, treatment of ob/ob mice with liraglutide for 2 weeks produced a significant decrease in CHOP expression in visceral adipocytes (**Figure 1C**). To ascertain whether GLP-1R activation directly regulates the ER stress response in adipocytes, we treated cultured mature rat adipocytes differentiated from SVF with thapsigargin (Tg), a pharmacological inducer of ER stress, for 16 hr in the presence or absence of GLP-1. Thapsigargin significantly increased the expression of CHOP and GLP-1 suppressed this increase in adipocytes (**Figure 1D** and **Supplementary Figure 1**). We also found thapsigargin suppressed insulin-stimulated Akt activation as evaluated by its phosphorylation at Ser 47. GLP-1 treatment reversed this suppression (**Figure 1D** and **Supplementary Figure 1**). Taken together, these data illustrate that GLP-1 attenuated the expression of CHOP, a molecular mediator of ER stress induced IR, in visceral fat cells of obese mice, thus GLP-1 might directly engaged in pathways modulating insulin signaling in ER-stressed adipocytes.

### GLP-1 Attenuates Insulin Resistance and Regulates Both the PERK and the IRE1/XBP-1 Arms of the UPR in Mouse Adipocyte Cell Line

**FIGURE 2:**
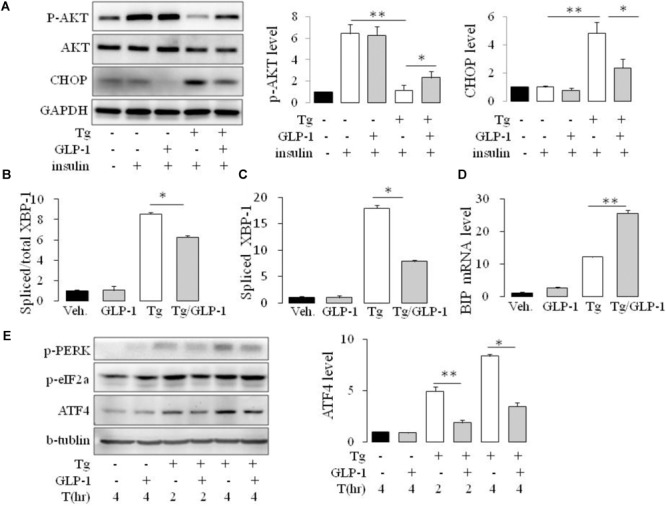
GLP-1 attenuated insulin resistance (IR) and regulated both PERK and IRE1/XBP-1 arm of the UPR in mouse adipose cell line. 3T3-L1 preadipocytes were induced to differentiate into mature adipocytes. **(A)** The differentiated adipocytes were fasted for 6 h in low-glucose DMEM media with 0.2% BSA (fasting media) and then were treated with vehicle alone or thapsigargin (Tg) in the absence or presence of 50 nM GLP-1 in fasting media for 16 h. The cells were then stimulated with insulin (100 nM) in fasting media for 10 min. **(B–D)** The differentiated adipocytes were fasted for 16 h in fasting media and then were treated with vehicle alone or thapsigargin (Tg) in the absence or presence of 50 nM GLP-1 in fating media for 9 h **(B–D)** or for 2–4 h **(E)**. Three wells of cell extracts were pooled together and were analyzed by immunoblotting for **(A)** phospho-AKT Ser47 (p-AKT), total AKT and CHOP, **(E)** ATF-4, P(Ser51)-eIF2a and P(Thr980)-PERK, or by quantitative PCR for **(B,C)** total and spliced XBP-1 and **(D)** BIP. GAPDH or b-tublin was used as internal control. Data represent means ± SE of four **(A)** or three **(B–E)** independent experiments. ^∗^*P* < 0.05 and ^∗∗^*P* < 0.01.

To ascertain whether GLP-1 treatment also affects the components of the UPR in adipocyte cell line, we treated cultured mature adipocytes differentiated from 3T3-L1 preadipocytes with pharmacological inducer of ER stress in the presence or absence of GLP-1. We found, thapsigargin (Tg) suppressed insulin-stimulated Akt activation as evaluated by its phosphorylation at Ser 47, and remarkably, treatment with GLP-1 attenuated this suppression (**Figure 2A** and **Supplementary Figure 2**). Development of ER stress response was assessed by measuring the level of CHOP and sXBP-1 which are indicators of activation of the PERK and IRE1 arms, respectively, of the UPR (Wu and Kaufman, 2006). We found that thapsigargin (Tg) increased the CHOP protein expression in a time- and dose- dependent manner (**Supplementary Figures 3A,B**). We got consistant result from another ER stress inducer tunicamycin (Tm) on the CHOP protein expression (**Supplementary Figures 3A–C**). Meanwhile, both thapsigargin and tunicamycin promoted the splice of XBP-1 (**Figures 2B,C** and **Supplementary Figure 3C**). GLP-1 markedly inhibited the magnitude of CHOP and sXBP-1 induction in the presence of thapsigargin or tunicamycin. Further experiments showed that the effects of GLP-1 on CHOP and sXBP-1 in ER-stressed adipocytes were also associated with increased expression of chaperone-encoding gene GRP78/BIP (**Figure 2D** and **Supplementary Figure 3C**). These results suggest that activation of GLP-1R signaling modifies the UPR in adipocytes via direct down-regulation of CHOP and sXBP-1 expression.

### GLP-1 Improves Insulin Signaling via Decreasing Expression of ATF4 and CHOP in ER-Stressed Adipocytes

**FIGURE 3:**
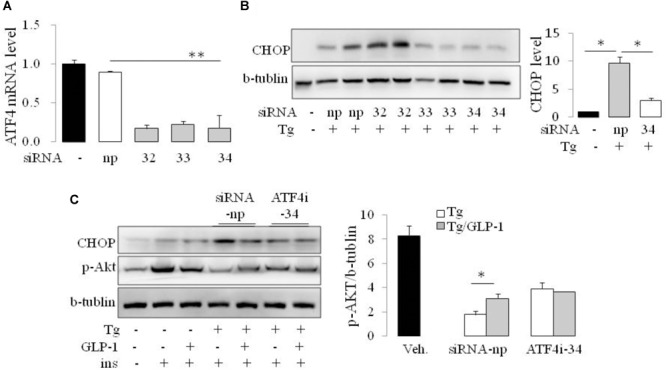
GLP-1 affected insulin signaling in ER-stressed adipocytes via ATF4. The adipocytes were transfected with non-specific (siRNA-np) or ATF4 specific siRNA fragments (ATF4i-32, 33, 34) to inhibit transcription of ATF4. Total cell extracts were then analyzed by quantitative PCR for ATF4 **(A)** and immunoblotting for CHOP **(B)**. Data represent means ± SE of images from the plot B and plot C. ^∗^*P* < 0.05. **(C)** 24 h after transfection with siRNA-np or ATF4i, cells were fasted for 6 h in low-glucose DMEM media with 0.2% BSA (fasting media) and then were treated with vehicle alone or thapsigargin (Tg) in the absence or presence of 50 nM GLP-1 in fasting media for 16 h. The cells were then stimulated with insulin (100 nM) in fasting media for 10 min. Three wells of cell extracts were pooled together and were analyzed by immunoblotting for phospho-AKT Ser47 (p-AKT), CHOP, and b-tublin (loading control). Data represent means ± SE of three independent experiments. ^∗^*P* < 0.05 and ^∗∗^*P* < 0.01.

As CHOP is transcriptionally induced during ER stress in response to PERK-eIF2α-mediated translation of ATF4, we examined whether GLP-1 regulates CHOP induction via modulation of the translation of ATF4. We found that thapsigargin (Tg) induced phosphorylation of PERK and eIF2α immediately, while ATF4 protein level increased gradually. GLP-1 decreased ATF4 protein expression associated with decreased levels of phospho-Thr980-PERK (p-PERK) and phospho-Ser51 eIF2α (p-eIF2α) (**Figure 2E**). To determine whether PERK arm of UPR has a role in regulating effect of GLP-1 on ER stress induced IR, we silenced the ATF4 expression with specific siRNA for ATF4 (**Figure 3A**). The expression level of CHOP, a target of ATF-4 transcriptional regulation, significantly decreased in the cells following knocking down of ATF4 (**Figure 3B**). Furthermore, in the cells lacking ATF-4, thapsigargin-induced inhibition on insulin-stimulated Akt activation was significantly attenuated. Moreover, GLP-1 lost the improvement effect on insulin signaling in ER-stressed cells with blocking the induction of ATF4/CHOP (**Figure 3C**). These observations implicate ATF4 as a potential intermediary conveying GLP-1 signaling to the PERK arm of UPR in regulating insulin signaling pathway.

### GLP-1 Decreases ATF4 Expression in ER-Stressed Adipocytes via Modulating mTOR Activity

**FIGURE 4:**
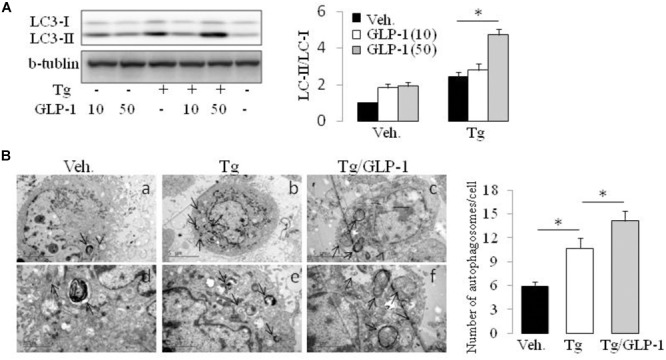
GLP-1 promoted the autophagy process activated by ER stress. The differentiated adipocytes fasted for 16 h in low-glucose DMEM media with 0.2% BSA (fasting media) and then were treated with vehicle alone or thapsigargin (Tg) in the absence or presence of GLP-1 for 4 h. **(A)** Three wells of cell extracts were pooled together and were then analyzed by immunoblotting for LC3. Conversion of LC3-I to LC3-II is qualified by calculating the intensities of Western blotting. Data represent means ± SE of three independent experiments. **(B)** Four wells of cells were pooled together for electron microscopic analysis. Panels d to f were high magnification images of panels a to c, respectively. Typical autophagosomes (arrows) are indicated. The numbers of autophagosomes in adipocytes were calculated in a total of 15 electron microscopical sections. Data represent means ± SE. ^∗^*P* < 0.05 and ^∗∗^*P* < 0.01.

Autophagy is a process by which cell components are degraded to maintain essential activity and viability in response to stress conditions, including ER stress-induced IR (Zhang et al., 2015). Thus, we investigated whether autophagy system has a role in the process of GLP-1 improved IR in the cells with ER stress. Conversion of MAP1LC3B from its cytosolic form (LC3-I) to its lipidated membrane-bound form (LC3II) is a key marker for autophagy. We found thapsigargin (Tg) incubation made an increase in LC3II/LC3I ratio, whereas treatment with GLP-1 enhanced the magnitude of LC3II/LC3I ratio (**Figure 4A**). The activation of autophagy also can be visualized by the formation of autophagosomes, which are recognized at the ultrastructural level as double-membrane vacuolar structures containing visible cytoplasmic contents. Consistently, we found the autophagosomes were also observed in cells incubated with thapsigargin and GLP-1 further increased the amount of autophagosome in the ER-stressed adipocytes (**Figure 4B**). The expression level of Atg7 and P62, genes involved in the autophagic process, was also found to be increased in response to ER stress, and further increased by GLP-1 (**Supplementary Figure 4**). These results suggested that GLP-1 regulates the autophagy process in ER-stressed adipocytes.

**FIGURE 5:**
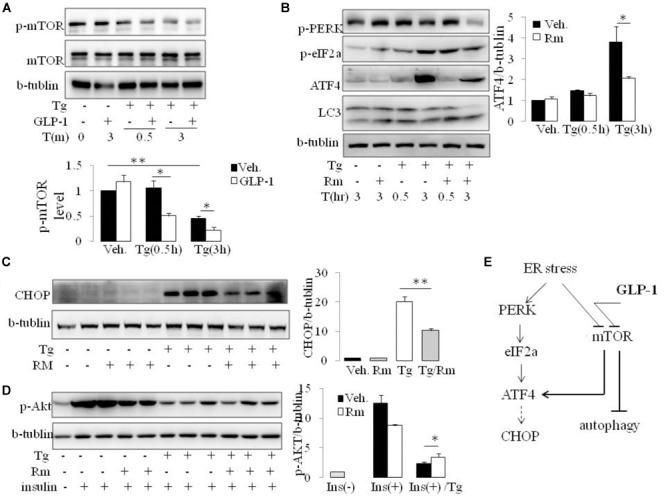
GLP-1 affects ATF4 translation induction in adipocytes following ER stress via preventing mTOR signaling pathway. **(A)** The differentiated adipocytes were fasted for 16 h in low-glucose DMEM media with 0.2% BSA (fasting media) and then were exposed to vehicle alone or thapsigargin (Tg) in the absence or presence of GLP-1 in fasting media for 0.5–3.0 h as indicated. Three wells of cell extracts were pooled together and were analyzed by immunoblotting for P(2448)-mTOR, mTOR, and b-tublin (loading control). Data represent means ± SE of four independent experiments. ^∗^*P* < 0.05 and ^∗∗^*P* < 0.01. Data for replication see **Supplementary Figure 5**. **(B)** The differentiated adipocytes were fasted for 16 h in fasting media and then were exposed to vehicle alone or thapsigargin (Tg) in the absence or presence of rapamycin (Rm) in fasting media for 0.5–3.0 h as indicated. Three wells of cell extracts were pooled together and were analyzed by immunoblotting for ATF-4, P(Ser51)-eIF2a, P(Thr980)-PERK, LC3 and b-tublin (loading control). Data represent means ± SE of three independent experiments. ^∗^*P* < 0.05. **(C,D)** The differentiated adipocytes were fasted for 6 h in fasting media and then were exposed to vehicle alone or thapsigargin (Tg) in the absence or presence of rapamycin (Rm) in fasting media for 16 h. Three wells of cell extracts were pooled together and were analyzed by immunoblotting for CHOP, phospho-AKT Ser47 (p-AKT) and b-tublin (loading control). Data represent means ± SE of three independent experiments. ^∗^*P* < 0.05 and ^∗∗^*P* < 0.01. **(E)** Model depicting the proposed mechanism of effects of GLP-1 on ER stress in adipocyte.

The mammalian target of rapamycin (mTOR), a kinase responsible for mitogen-induced cell proliferation/survival signaling, regulates the mammalian autophagy initiation by direct phosphorylation of Ulk1 (Kim et al., 2011; Shang and Wang, 2011). We found that ER stress induced by thapsigargin immediately decreased the phosphorylation of mTOR. Interestingly, in the presence of GLP-1, the phosphorylation level of mTOR was further decreased, and this effect took place at the early stage of ER stress (**Figure 5A** and **Supplementary Figure 5**). Since ATF4 was also reported to regulate autophagy in response to ER stress (Rzymski et al., 2010), we questioned whether the inhibition effect by GLP-1 on ATF4 expression during ER stress was mediated by modulating the activity of mTOR. Rapamycin (Rm), a macrolide antibiotic and immunosuppressive compound, is a typical inhibitor of mTOR signaling. In the presence of Rm, translation of ATF4 by thapsigargin was significantly inhibited, and this was associated with decreased PERK phosphorylation (**Figure 5B**). Subsequently, treatment with Rm significantly reduced the expression level of CHOP in the ER-stressed cells (**Figure 5C**). Furthermore, the inhibitory effect of thapsigargin on insulin-induced Akt phosphorylation was significantly attenuated by rapamycin (**Figure 5D**). Notably, the pattern of the effect on ATF4 through PERK arm of UPR pathway by rapamycin is similar to that by GLP-1.

Rapamycin is reported have effect on ER stress in an autophagy-dependent manner (Yin et al., 2015). However, inhibiting autophagy with wortomannin (WM) and chloroquine (CQ), ATF4 induction was abolished under ER stress in the presence and absence of GLP-1, despiting increase in p-PERK and p-eIF2a level (**Supplementary Figure 6**). Thus, our results suggest that different from the effect partern of Rapamycin, GLP-1’s action on ER stress is associated with but not dependent on autophagy process. It is likely that GLP-1 targets mTOR to help cells recover from overwhelming stress situation via multiple pathways, especially via attenuating UPR and initiating autophagy (**Figure 5E**).

## Discussion

The epidemic evidences of worldwide rapid rise in prevalence of obesity and diabetes have focused efforts on development of intervention strategies which both sensitize insulin action and produce sustained improvements in β cell function (Rhodes, 2005). GLP-1 stimulates insulin biosynthesis and secretion and maintains β cell hemostasis through promotion of β cell proliferation and inhibition of β cell death (Kumar et al., 2007; Soltani et al., 2007; Wang et al., 2010). GLP-1 also exerts a variety of biological actions independent of islet β cells, including reduction in gastric emptying, suppression of glucagon secretion and body weight sparing effects. Although controversial, previous studies showed that GLP-1 can also improve peripheral insulin sensitivity (Liu et al., 2014, 2016). Here, we demonstrate that GLP-1 improves insulin sensitivity of ER-stressed adipocyte through modulation of UPR.

Adipocyte plays a central role in obesity-associated IR (Bergman and Mittelman, 1998). Chronic overfeeding induces ER stress in adipocyte. In addition to the direct inhibitory effect on insulin signaling, UPR also activates inflammatory pathway in adipocyte, which initial adipose macrophage infiltration, resulting in further impairment of insulin signaling in stressed adipocytes. Furthermore, insulin-resistant adipocytes, being more lipolytic and less liposynthetic, increase circulating level of free fatty acids (FFAs). Elevated circulating FFA plus chemokines and cytokines, likely contributed by both inflamed adipose tissue and blood mononuclear cells, causes IR in other organs such as liver and muscle (Lai et al., 2008).

Approaches aimed to reduce ER stress is effective on improving insulin sensitivity in both animal models and human beings (Hirosumi et al., 2002). By enhancing ER folding capacities with chemical chaperones, obese and insulin resistant ob/ob mice showed dramatically improved glucose tolerance and systemic insulin sensitivity without affecting body weight (Ozcan et al., 2006). Diabetic mice overexpression the ER chaperone ORP150 displayed improved glucose tolerance and enhanced insulin signaling in liver and muscle (Ozawa et al., 2005). Targeting UPR-related molecules also protected cells from ER stress induced cell death *in vitro* and importantly *in vivo* in rat (Boyce et al., 2005). Intriguingly, two chemicals in use or in clinical trials for the treatment of type 2 diabetes have also been shown to have effects on UPR pathway molecules. Thiazolidinediones, known agonists of PPARγ, were shown to phosphorylate eIF2α and inhibit protein synthesis independent of PPARγ (Palakurthi et al., 2001). Second, the anti-inflammatory salicylates also led to increased eIF2α phosphorylation via PERK activation (Silva et al., 2007). In the present study, we demonstrate that GLP-1 decreased CHOP expression leading to improved insulin signaling in ER stressed adipocytes. Thus, it is likely that the beneficial effects of GLP-1 on systemic glucose homeostasis in obesity and obesity related diabetes may be related to its ability to protect/maintain ER function of hypertrophic adipocytes.

When cell is exposed to external or internal cues, multiple processes are activated to restore the cellar homeostasis or committ to cell death, including the UPR, autophagy and mitochondrial function. While these processes trigger different cellar responses, there is also widespread crosstalk (Senft and Ronai, 2015). When unfolded or misfolded proteins accumulate in the ER, autophagy is designed to be stimulated as a degradation system to clean up unnecessary proteins. On one hand, PERK and Ire1a arms of UPR could activate autophagy process (B’chir et al., 2013). On the other hand, preactivation of autophagy increased molecular chaperones to reduce excessive ER stress (Sheng et al., 2012). While, disturbance of autophagy rendered cells vulnerable to ER stress induced cell death. In the present study, we found that ER stress immediately induced the activation of autophagy evidenced by LC3 conversion and autophagosome formation in adipocytes. Furthermore, inducing autophagy significantly suppressed ATF4 expression in ER-stressed cells. However, blocking autophagic flux did not increase ATF4 level under ER stress condition with or without GLP-1. Thus, our work does not support that the effect of GLP-1 on ER stress is through activation of autophagy. Actually, GLP-1 inhibited p-mTOR, substrate of rapamycin, to preventing the ER stress induced ATF4 expression, which activated autophagy in parallel.

GLP-1 improves insulin sensitivity via multiple ways. Many studies have suggested that the effect of GLP-1 on insulin sensitivity is attributable to the suppression of weight gain. However, Gastaldelli et al. (2016) studied the acute effects of exenatide on adipose tissue in human subjects with IR. They found that GLP-1 receptor agonists had direct effects on insulin signaling during a glucose load. Their results also suggest that the cross-talk between adipose tissue and liver was important for the insulin improving effects of exenatide. Brain GLP-1 signaling also improved whole body insulin sensitivity in a GLP-1R dependent manner (Cabou et al., 2011). Inflammation plays an important role in the obesity-induced IR (Shoelson et al., 2006). GLP-1 reduced macrophage infiltration in adipose tissue and directly inhibited inflammatory pathways in adipocytes contributing to the improvement of insulin sensitivity (Lee et al., 2012). The complexity of the effects of GLP-1 on insulin sensitivity makes it much important to figure out the direct effects on insulin responsive cells which helps develop clinical practice guideline for GLP-1R agonists.

In summary, the present study reveals two key findings. First, GLP-1 improved IR in ER stressed adipocytes by modulating PERK pathway via targeting ATF4/CHOP expression, which deepens our understanding of biology of GLP-1. Secondly, we demonstrated mTOR as an important pathway mediating the progression of UPR in ER stressed adipocytes. These results refer GLP-1R agonists and mTOR pathway as potential targets for the treatment of obesity or diabetes associated IR.

## Author Contributions

YJ and ZW conducted the experiments and data analysis. BM performed the data analysis and interpretation, and revised the manusript. LF and NY conducted the animal experiments. BL and QW edited the manuscript. RL conducted study design, data analysis and interpretation, manuscript composition and manuscript editing.

## Conflict of Interest Statement

The authors declare that the research was conducted in the absence of any commercial or financial relationships that could be construed as a potential conflict of interest.
