# Supplementary material for: GLP-1 Improves Adipocyte Insulin Sensitivity Following Induction of Endoplasmic Reticulum Stress
Source: Front Pharmacol. 2018 Oct 16;9:1168. doi: 10.3389/fphar.2018.01168 (PMC6232689; doi:10.3389/fphar.2018.01168)
Supplement: Supplementary file 1 [file Data_Sheet_1.PDF]

Independent replication for Figure 1D

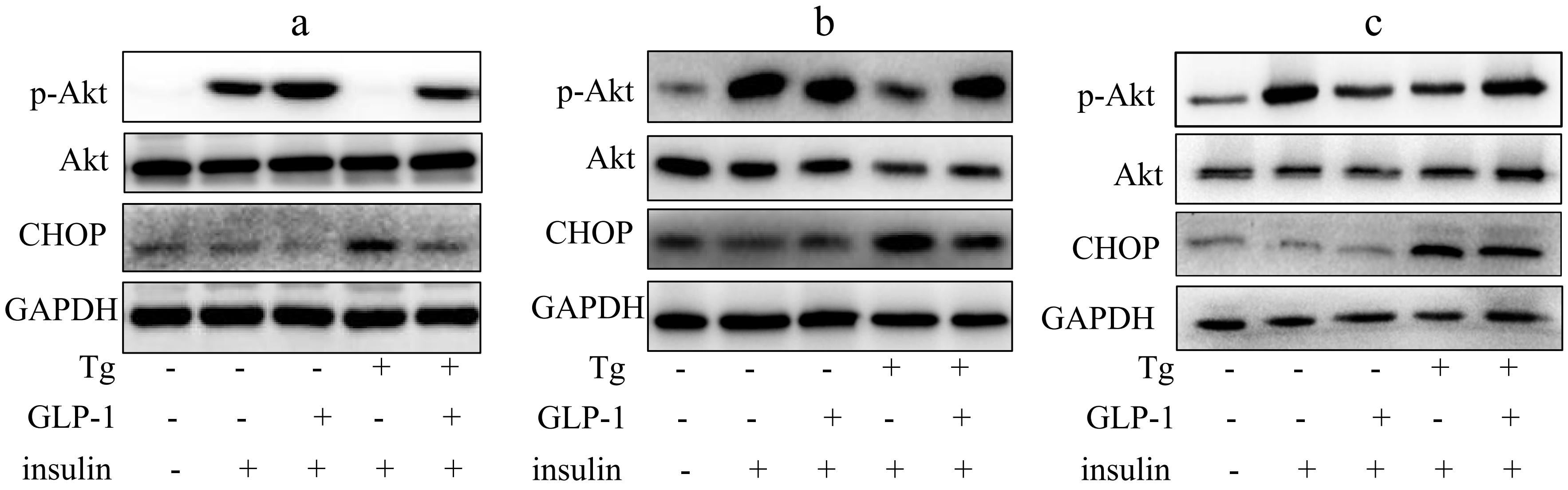

Sup Fig. 1

Independent replication for Figure 2A

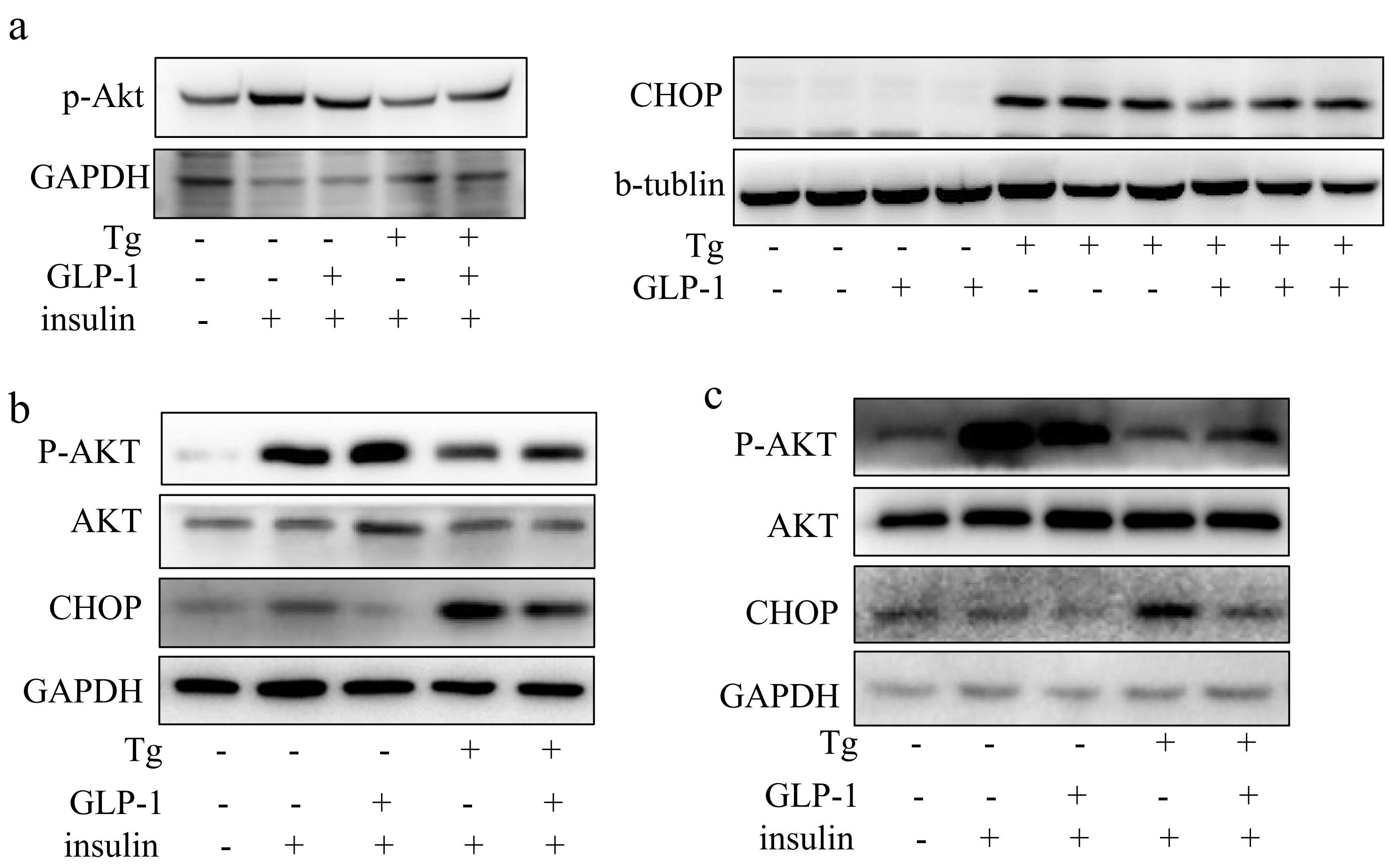

Sup Fig. 2

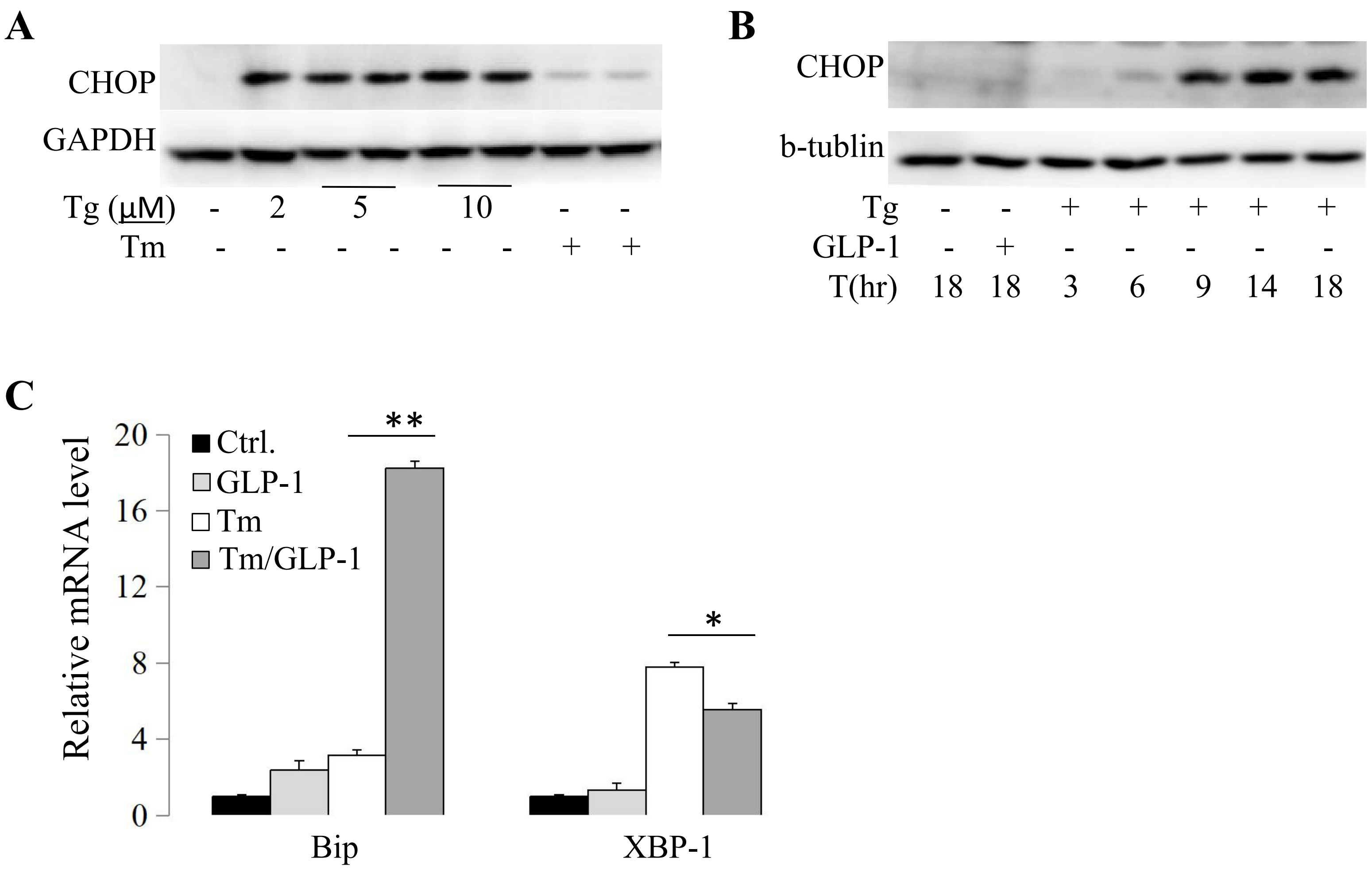
A
CHOP
GAPDH
Tg (μM) - 2 5 10 - -
Tm - - - - - - + +
B
CHOP
b-tublin
Tg - - + + + + +
GLP-1 - + - - - - -
T(hr) 18 18 3 6 9 14 18
C
Relative mRNA level
20
16
12
8
4
0
Ctrl.
GLP-1
Tm
Tm/GLP-1
**
*
Bip
XBP-1

Sup Fig. 3

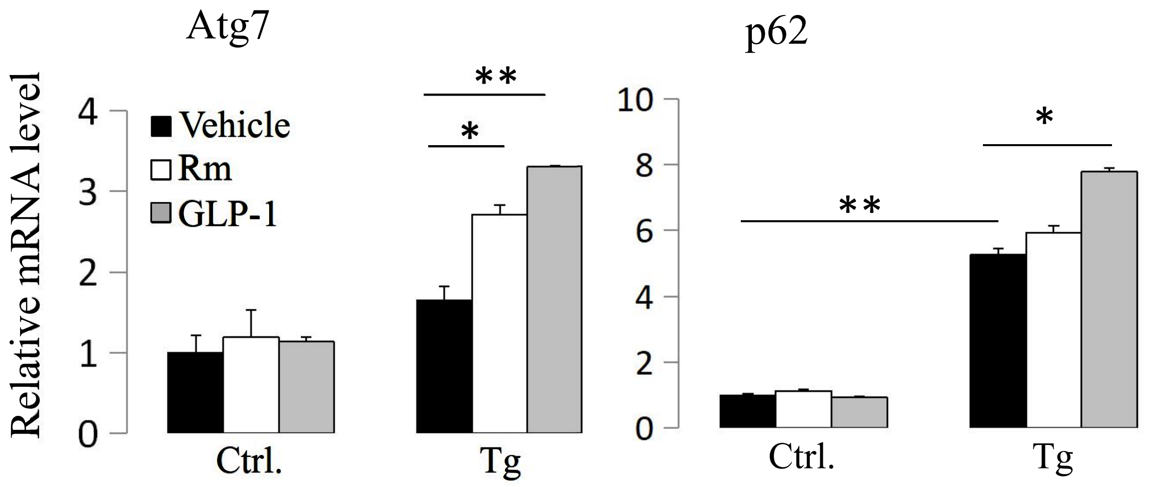

Atg7
p62
Relative mRNA level
Vehicle
Rm
GLP-1
**
*
0
1
2
3
4
0
2
4
6
8
10
Ctrl.
Tg
Ctrl.
Tg

Sup Fig. 4

Independent replication for Figure 5A

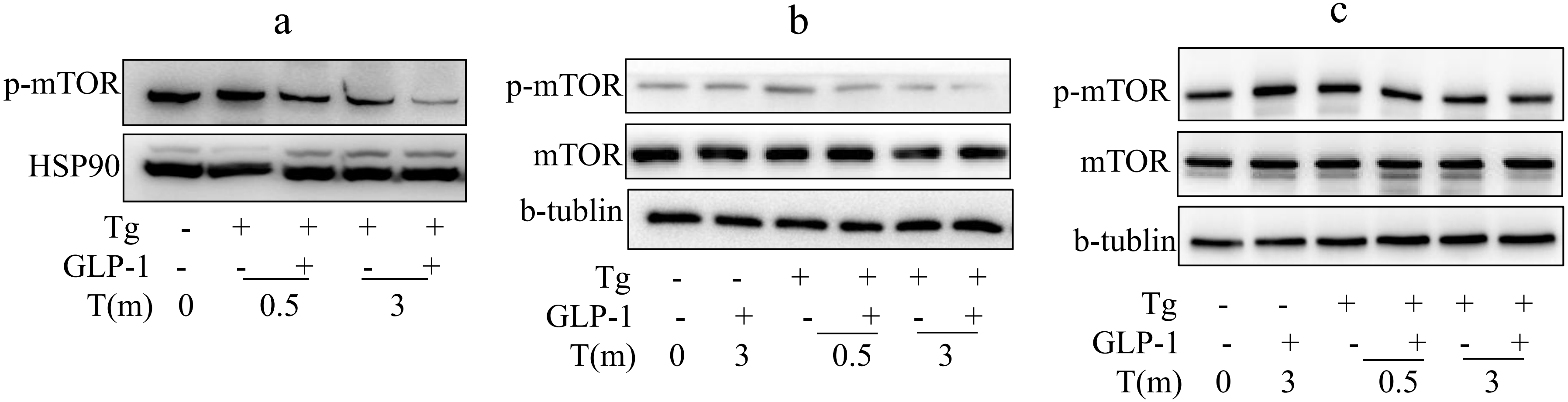

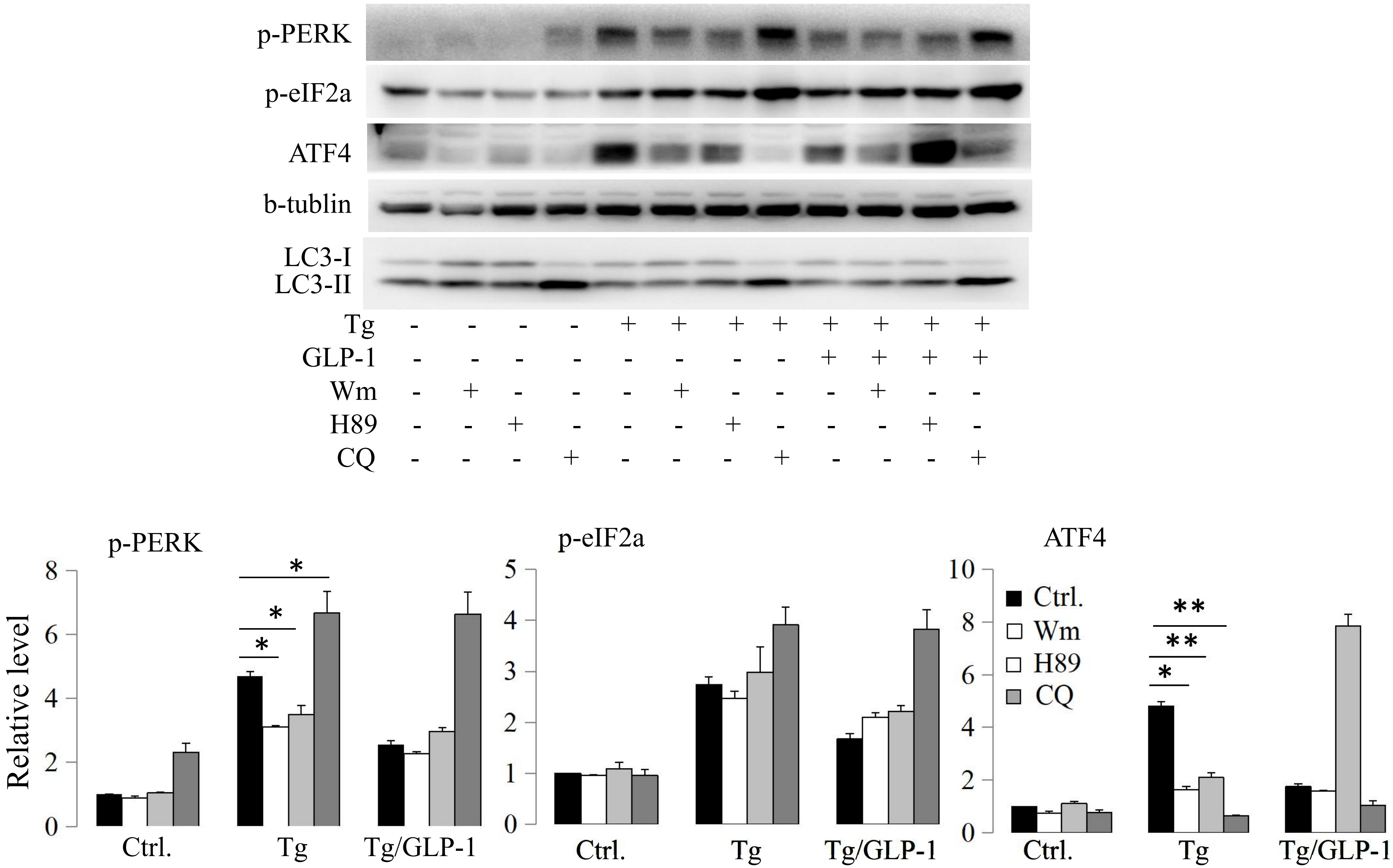

p-PERK
p-eIF2a
ATF4
b-tublin
LC3-I
LC3-II
Tg - - - - + + + + + + + +
GLP-1 - - - - - - - - + + + +
Wm - + - - - + - - - + - -
H89 - - + - - - + - - - + -
CQ - - - + - - - + - - - +
p-PERK
p-eIF2a
ATF4
Relative level
Ctrl.
Wm
H89
CQ
Ctrl.
Tg
Tg/GLP-1

Sup Fig. 6
