# Supplementary material for: GLP-1 Improves Adipocyte Insulin Sensitivity Following Induction of Endoplasmic Reticulum Stress
Source: Front Pharmacol. 2018 Oct 16;9:1168. doi: 10.3389/fphar.2018.01168 (PMC6232689; doi:10.3389/fphar.2018.01168)
Supplement: Supplementary file 2 [file Data_Sheet_2.PDF]

Supplementary figure legends:

Supplementary Figure 1. Independent replication for figure 1D.

Supplementary Figure 2. Independent replication for figure 2A.

Supplementary Figure 3. 3T3-L1 preadipocytes were induced to differentiate into mature adipocytes. The differentiated adipocytes were fasted for 6 hrs in low-glucose DMEM media with 0.2% BSA (fasting media) and then were treated with vehicle alone or thapsigargin (Tg) tunicamycin (Tm) in fasting media for 16 hrs with indicated doses (A) or for the indicated periods of time (B). 3 wells of cell extracts were pooled together and were analyzed by immunoblotting for CHOP and GAPDH or β-tublin (loading control). (C) Cells were fasted for 6 hrs in fasting media and then were treated with vehicle alone or tunicamycin (Tm) in the absence or presence of 50 nM GLP-1 in fasting media for 9 hrs. 3 wells of cell extracts were pooled together and were analyzed by quantitative PCR for XBP-1 and BIP. Data represent means ± SE of three independent experiments. ***P*<0.01, **P*<0.05.

Supplementary Figure 4. 3T3-L1 preadipocytes were induced to differentiate into mature adipocytes. Cells were fasted for 6 hrs in low-glucose DMEM media with 0.2% BSA (fasting media) and then were treated with vehicle alone or thapsigargin (Tg) in the absence or presence of GLP-1 or rapamycin (Rm) in fasting media for 9 hrs. 3 wells of cell extracts were pooled together and were analyzed by quantitative PCR for Atg7 and p62. Data represent means ± SE of three independent experiments. ***P*<0.01, **P*<0.05.

Supplementary Figure 5. Independent replication for figure 5A.

Supplementary Figure 6. 3T3-L1 preadipocytes were induced to differentiate into mature adipocytes. Cells were fasted for 4 hrs in low-glucose DMEM media with 0.2% BSA (fasting media) and then were pre-treated with wortmannin (Wm, a PI3K

inhibitor), H89 (a PKA inhibitor) or chloroquine (CQ, an autophagy flow inhibitor) in fasting media for 30 min. Finally, cells were treated with vehicle alone or thapsigargin (Tg) in the absence or presence of GLP-1, Wm, H89 or CQ in fasting media for 2 hrs. 3 wells of cell extracts were pooled together and were analyzed by immunoblotting for ATF-4, P(Ser51)-eIF2a, P(Thr980)-PERK, LC3 and β-tublin (loading control). Data represent means ± SE of three independent experiments. $^{**}P<0.01$, $^{*}P<0.05$.
